# The positive solitude scale (PS): psychometric properties among Chinese older

**DOI:** 10.1186/s12889-024-19237-8

**Published:** 2024-08-09

**Authors:** Zhiguang Fan, Huilin Cai, Xiaoli Shi, Ningyao Yu, Lei Chen

**Affiliations:** 1 Department of Psychology, Shaoxing University, Shaoxing, 312000 People’s Republic of China; 2 School of English, Jilin International Studies University, Changchun, 130117 People’s Republic of China; 3 School of Education, Jilin International Studies University, Changchun, 130117 People’s Republic of China; 4 Department of Marxism, Changchun University of Chinese Medicine, Boshuo Street 1035, Changchun City, Jilin Province 130117 People’s Republic of China

**Keywords:** Older people, Positive solitude, Reliability, Validity

## Abstract

**Background:**

Positive solitude, taken as a meaningful activity, contributes to the improvement of health, well-being, and quality of life of older adults. The purpose of this study was to examine the reliability and validity of the Positive Solitude Scale (PS) among Chinese older to provide a reference for related research.

**Methods:**

A convenience sample of 608 older people from 10 provinces in China was used to conduct the survey.

**Results:**

The Chinese version of the PS consisted of 9 items with a unidimensional structure, which could explain 60.91% of the variance. The factor loadings of each item ranged from 0.67 to 0.82, and the communality ranged from 0.44 to 0.68. The confirmatory factor analysis showed good model fit (χ^2^/df = 2.771, RMSEA = 0.076, CFI = 0.972, IFI = 0.972, TLI = 0.959, PNFI = 0.665, PCFI = 0.675). It was found from the criterion-related validity test that PS scores were significantly and positively correlated with Satisfaction With Life Scale (SWLS), Autonomy, Competence, and Relatedness scores (*r* = 0.45 to 0.44); PS scores were significantly and negatively correlated with Short-Form UCLA Loneliness Scale (ULS-6), Kessler Psychological Distress Scale (K10), Ego Depletion Scale (EDS), and Acceptance and Action Questionnaire-2nd Edition (AAQ-II) (*r* = -0.27 to -0.36). The Cronbach’s α coefficient value for the scale was 0.917; the split-half reliability coefficient value was 0.928. In addition, the PS showed cross-gender consistency.

**Conclusions:**

The PS presented favorable psychometric characteristics in older people, which can be used as a valid tool for assessing older people’s positive solitude.

## Introduction

For anyone, maintaining good relationships and social connections is a necessary condition for healthy growth [1]. However, this does not mean that individuals always want to be in social interaction, instead, they still have the need for solitude, which, as a developmental need, is a prerequisite for human growth [2]. Solitude is often defined as the physical state of being alone or psychological distance from others [3]. Solitude is common throughout the life cycle, even in childhood [4]. In different age groups, individual needs for solitude and its functions are different [5]. Older people spend more time alone than younger people [6]. It is found in a smartphone-based event-contingent ambulatory assessment study that older people spend about 39 min a day on social interaction, while the time they spend in solitude is as much as five hours [7]. In addition, older people crave and seek solitude more and enjoy the positive effects of being alone [8]. However, caregivers of older adults tend to view their solitary behavior as an escape and passive choice, and intentionally limit their experience of solitude [9].

Solitude is seen as a double-edged sword, both a threat to the development and health of individuals and a potential benefit. In a review study, it was noted that the effects of solitude on the well-being of older people are complex, with both positive and negative effects [10]. The individual’s intrinsic motivation and preference can affect how solitude comes into play [11]. For instance, when individuals choose to be alone out of the intrinsic motivation of self-determination, they can often gain positive emotions and have a favorable impact on their development [12]. To better examine the value and significance of solitude, the researchers proposed the concept of positive solitude, which means when, where, and how an individual engages in meaningful, enjoyable activity or experience conducted by himself, and has a positive impact on their quality of life [13]. It is worth noting that positive solitude can occur regardless of the presence or absence of other people and the external environment, even in a busy cafe, restaurant, or playground.

Solitude, as a fundamental experience of human life, is also one of the fundamental determinations of our being [14]. In most philosophies and religions, the value of positive solitude in promoting individual spirituality, insight, and self-reflection is valued, and is seen as an important way to seek meaning and well-being in life [15–17]. For example, Yoga and Buddhism, which originated in India, as well as Taoism in China, promote positive solitude and develop practices such as sitting still, mindfulness meditation, internalization, and samadhi. Christian and Catholic ascetic contemplatives will also actively reduce or avoid social contact to focus more on their own spiritual world and achieve intimate conversation with God [18]. In addition, studies in the field of psychology have found that positive solitude can improve individual creativity, self-reflection, identity development, and social self-efficacy [19, 20]. For older adults, positive solitude helps them better adapt to the dwindling social network, thus improving well-being [21]. Previous studies have found that positive solitude is remarkably positively correlated with life satisfaction, autonomy, self-acceptance, personal growth and purpose, friendship quality, and social interaction level of the older population, and on the other hand, it is remarkably negatively correlated with psychological distress and loneliness [22]. Positive solitude, as a meaningful activity, is therefore of positive significance for improving the health, well-being, and quality of life of older adults.

To measure the construct of solitude, researchers have developed corresponding scales under different theoretical frameworks. The preference for solitude scale (PSS), consisting of 12 items, was developed by Burger et al. to measure individual preference for solitude [23]. PSS, taking a forced-choice approach, requires individuals to determine whether they prefer to be alone or with others in different scenarios. However, PSS does not involve a motivational component, and individuals may choose to be alone for passive reasons, which is not consistent with the concept of positive solitude. In addition, different researchers dispute the dimension division of PSS. For example, Burger et al. extracted 4 factors in the exploratory factor analysis of PSS, but the interpretation rate of the first factor was more than half. Therefore, he believed that PSS should be of a unidimensional structure. However, Cramer et al. found in their study that PSS is composed of 3 factors, namely, Need for Solitude, Enjoyment of Solitude, and Productivity During Solitude [24].

In previous quantitative studies on solitude, some of the tools developed reflect the content of positive solitude. The Solitude Questionnaire (SQ), consisting of 9 items, is divided into 3 dimensions: inner-directed solitude, loneliness, and outer-directed solitude, which can measure the positive, negative, and neutral results from solitude [25]. In addition, Coplan et al. developed the Solitude and Aloneliness Scale (SolAS), consisting of 12 items, to assess an individual’s satisfaction with solitude time [26]. Another commonly used scale is the 14-item Motivation for Solitude Scale - Short Form (MSS-SF) developed by Thomas et al. It is divided into 2 dimensions: Self-determined solitude (SDS) and Not self-determined solitude (NSDS) [27]. MSS-SF was primarily used to measure an individual’s motivation for choosing to be alone, such as a desire for contemplation, self-reflection, social anxiety, or rejection by peers. It should be noted that SQ, SolAS, and MSS-SF were not developed specifically to evaluate positive solitude, but only covered positive solitude in some items or dimensions. At the same time, the above 3 scales were developed for adolescents and early adulthood, and their applicability in older age groups is unclear.

Considering that there are significant differences in the time, manner, content, and impact of solitude on the lives of people of different ages, some researchers have developed scales suitable for specific populations [28]. For example, Galanak et al. developed the Children’s Solitude Scale (CSS) composed of 4 dimensions: Self-Reflection, Autonomy/Privacy, Activities, and Concentration [29]. CSS, consisting of 45 items, takes a long time to respond and is not suitable for rapid screening on a large scale. Meanwhile, the results of the confirmatory factor analysis show that the model fitting index of CSS is poor. In addition, Coplan et al. developed the Child Social Preference Scale (CSPS) to measure the social motivation of children playing with their peers [30]. CSPS is a nurse-administered rating scale with 11 items in 2 dimensions, namely Conflicted Shyness and Social Disinterest. According to the analysis of the existing literature, previous studies have developed corresponding scales around the status, preference, motivation, and outcome of solitude. At present, however, there is a lack of specific scales to measure the psychological trait of positive solitude. At the same time, positive solitude is an important part of the daily activities for older people and has a significant impact on their health and quality of life [31]. However, of the assessment tools available, fewer involve older persons. To this end, a specific scale is needed to assess the positive solitude in older adults.

To measure the construct of positive solitude, Palgi et al. developed the Positive Solitude Scale (PS) applicable to Israeli adults aged over 40, especially older people under the theoretical framework of positive psychology [32]. PS consists of 9 items and has good face validity, structural validity, and test-retest reliability. As an effective tool specifically used to evaluate positive solitude, whether PS has certain cross-cultural applicability needs to be verified. Culture can influence people’s judgments about the value and meaning of interpersonal relationships. Individuals with different cultural orientations have different attitudes toward solitude [8]. In cross-cultural studies, it is found that compared with collectivist cultures, people in individualistic cultures are more negative about solitude [33]. For example, Jiang et al. found that older adults in China are more receptive to solitude than older people in Canada, and can experience more positive emotions and less negative emotions when they are alone [34]. In the study of Maes et al., it is also found that in comparison to the Belgian, Chinese people with collectivist values have a more positive attitude towards solitude [35].

Currently, two studies have examined the applicability of PS in Chinese culture. In the study of Yu et al., PS was translated into Chinese and the validity of the scale was tested in young and middle-aged population in China [36]. However, this study did not strictly follow the revision steps of the scale, and only conducted exploratory factor analysis and confirmatory factor analysis, without examining other reliability and validity indicators. In another study, researchers tested the validity of PS in a population of Chinese college students [37], and found that the Chinese version of PS was consistent with the original scale in terms of the number of items and dimension division, and showed satisfactory psychometric properties. Both of the above studies were conducted among college students and middle-aged individuals, and did not involve older adults. Therefore, the applicability of PS in older people in China still needs to be further verified.

The purpose of this study was to examine the validity of the Chinese version of PS in older population in China, so as to provide a reference for the relevant research on positive solitude in older adults. It was hypothesized that the Chinese version of PS has good reliability and validity and can be used as an effective tool to evaluate positive solitude in Chinese older people. This study further investigated the criterion-related validity of the Chinese version of PS. Positive solitude is considered one of the predictors of mental health [32]. Previous studies have found that positive solitude can enhance an individual’s sense of control and autonomy, improve stress coping and life satisfaction, and reduce negative emotional experiences of anxiety and depression [25, 38]. In addition, previous studies have found that Vipassana Meditation [39] and Mindfulness Meditation Practice [17] can help improve individuals’ cognitive flexibility and positive solitude, and promote healthy mental development.

To this end, The short-form UCLA Loneliness Scale (ULS-6), The Satisfaction With Life Scale (SWLS), The Needs Satisfaction Scale (NSS), The Kessler Psychological Distress Scale (K10), The Ego Depletion Scale (EDS), and The Acceptance and Action Questionnaire-2nd Edition (AAQ-II) were selected as criterion tools. The study hypothesized that the scores of PS were remarkably negatively correlated with those of ULS-6, K10, EDS, and AAQ-II (to measure individual’s experiential avoidance, not acceptance and action), and remarkably positively correlated with those of SWLS and NSS. At the same time, considering the potential gender differences in positive solitude, the study further examined the cross-gender invariance of PS [40].

## Method

### Procedure and participants

The minimum sample size required was calculated before the survey. In order to ensure the stability of the results of factor analysis, it is suggested that the minimum sample size should be 10 times or even 20 times that of the items [41]. In the current study, 20 times is used as the calculation standard. The Positive Solitude Scale (PS) includes 9 items, so the minimum sample size should be 180 people. In addition, according to the criteria of the National Bureau of Statistics of China for the age of older adults, people aged 65 and above were selected as the participants. The survey was completed on a one-to-one basis by the researchers. The anonymity of the study and the confidentiality of the data were explained to the respondents in detail before the survey. After obtaining the informed consent of the participants, the investigators sent the respondents a link to the online survey and the respondents completed the answers independently. As some older people were not familiar with electronic devices, the investigators explained how to start answering and how to submit the questionnaire. The study followed the Declaration of Helsinki and has been approved by the Ethics Committee of Jilin International Studies University (Approval number: JY202211003).

The questionnaire survey was conducted by means of convenient sampling. The investigators are made up of both undergraduate and graduate students who have received uniform training, and they used the summer vacation to survey older adults in their hometown. The survey covers a total of 10 provinces of China, including Zhejiang, Shandong, Fujian, Jiangsu, Guangdong, Jiangxi, Hubei, Henan, Guizhou, and Sichuan. The survey was conducted in two phases. The data from the first phase were used for item analysis (IA) and exploratory factor analysis (EFA), and the data from the second phase were used for confirmatory factor analysis (CFA), criterion validity, reliability, and cross-group invariance test.

A total of 302 older people were surveyed in the first phase, of whom 146 were male (48.34%) and 156 were female (51.66%); marital status of unmarried 5 (1.66%), married 249 (82.45%), divorced 9 (2.98%), and widowed 39 (12.91%); Among them, 296 (98.01%) were Han and 6 (1.99%) were ethnic minorities, with 160 (52.98%) living in urban areas and 142 (47.02%) in rural areas. In terms of health status, 274 persons (90.73%) do not have disabilities and 28 (9.27%) disabled; 148 (49.01%) of them graduated from primary school or below, junior high school 83 (27.48%), senior high school 32 (10.60%), college 14 (4.64%), undergraduate 19 (6.29%), and graduate or above 6 (1.99%); the minimum age is 65 years old, while the maximum is 92 years old, with an average age of 72.48 years old (SD = 5.61).

A total of 306 older people were surveyed in the second phase, of whom 126 were male (41.18%) and 180 were female (58.82%); marital status of unmarried 12 (3.92%), married 241 (78.76%), divorced 8 (2.61%), and widowed 45 (14.71%); Among them, 294 (96.08%) were Han and 12 (3.92%) were ethnic minorities, with 139 (45.42%) living in urban areas and 167 (54.58%) in rural areas. In terms of health status, 275 (89.87%) of them do not have disabilities and 31 disabled (10.13%); 172 (56.21%) of them graduated from primary school or below, junior high school 81 (26.47%), senior high school 28 (9.15%), college 10 (3.27%), undergraduate 8 (2.61%), and graduate or above 7 (2.29%); the minimum age is 65 years old, while the maximum is 91 years old, with an average age of 72.55 years old (SD = 5.50).

### Instrument

#### Positive solitude scale (PS)

PS is applicable to measure an individual’s attitude and tendency toward positive solitude [32], which consists of 9 items with a unidimensional structure and is rated on a 5-point scale ranging from 1 (not at all) to 5 (most of the time). The Chinese version of PS is intended for use in college students and early adulthood and shows good psychometric characteristics [36, 37]. The sum of the scores of each item is the total score. The higher the score, the more positive the attitude of older adults towards positive solitude, and the more inclined they are to choose positive solitude.

#### Short-form UCLA loneliness scale (ULS-6)

The UCLA Loneliness Scale is currently the most commonly used scale for measuring loneliness, and different versions have been developed. Among them, ULS-6, consisting of 6 items with a unidimensional structure, is a simple and valid tool for measuring loneliness in older adults [42], which is rated on a 4-point scale ranging from 1 (never) to 4 (often). The validity of Chinese version of ULS-6 was verified among Chinese older people [43]. Except for item 2, which is scored in reverse, the other items are scored in forward. The sum of the scores for each item is the total score, and the higher the score, the stronger the loneliness of older adults. In this study, the Cronbach’s α coefficient value of ULS-6 was 0.81.

#### Satisfaction with life scale (SWLS)

SWLS, consisting of 5 items with a unidimensional structure, can be used to measure individuals’ cognition, judgment, and satisfaction with their daily living conditions, which is rated on a 7-point scale ranging from 1 (strongly disagree) to 7 (strongly agree) [44]. The Chinese version of SWLS is widely used as the most commonly used measurement to assess the life satisfaction of Chinese of different ages, and has good reliability and validity [45]. The sum of the scores of each item is the total score, and the higher the score, the higher the life satisfaction level of older adults. In this study, the Cronbach’s α coefficient value of SWLS was 0.92.

#### Need satisfaction scale (NSS)

NSS was developed based on Self-determination Theory (SDT) and can be used to measure the satisfaction of an individual’s basic psychological needs [46, 47]. Yang et al. found that the Chinese version of NSS shows favorable factorial validity, criterion validity, and satisfactory internal reliability [48]. NSS consists of 9 items divided into 3 dimensions: autonomy, competence, and relatedness, and is rated on a 7-point scale ranging from 1 (very little) to 7 (very much). The sum of items in each dimension is the total score, and the higher the score, the higher the satisfaction degree of psychological needs of older adults. In this study, the Cronbach’s α coefficient values of autonomy, competence, and relatedness are 0.87, 0.90, and 0.91, respectively.

#### Kessler psychological distress scale (K10)

K10 is one of the most commonly used tools to measure an individual’s mental health status and is widely used in people of different ages [49]. The Chinese version of K10 consists of 10 items, divided into 2 dimensions of anxiety and depression, which are rated on a 5-point scale ranging from 1 (none of the time) to 5 (all of the time) [50]. The sum of the scores for each item is the total score, and the higher the score, the worse the mental health of older adults. In this study, the Cronbach’s α coefficient value of K10 was 0.95.

#### Ego depletion scale (EDS)

EDS, consisting of 5 items with a unidimensional structure, can measure the consumption of self-control resources after an individual experiences stressful events or negative life events [51], which is rated on a 5-point scale ranging from 1 (not at all) to 5 (very much). The Chinese version of EDS shows favorable validity and reliability [52]. The sum of the scores of each item is the total score, and the higher the score, the more serious the self-depletion of older adults. The Cronbach’s α coefficient value of EDS in this study was 0.90.

### Acceptance and action questionnaire-2nd edition (AAQ-II)

Experiential avoidance (EA) has been identified as a risk factor for mental health and can lead to psychological inflexibility. AAQ-II is the most commonly used tool for evaluating EA. AAQ-II, which is rated on a 7-point scale ranging from 1 (never true) to 7 (always true), is a unidimensional structured scale consisting of 7 items [53]. The Chinese version of AAQ-II is an effective measurement tool to test Chinese people’s EA [54]. The sum of the scores of each item is the total score, and the higher the score, the higher the degree of EA of older adults. The Cronbach’s α coefficient value of AAQ-II in this study was 0.92.

### Statistical analysis

The study utilized SPSS 24.0 to perform item analysis and EFA on the data from the first phase. The main purpose of item analysis is to examine the differentiation and the homogeneity of each item. To this end, 3 methods, namely the critical ratio value method, total correlation, and Cronbach’s α coefficient test, were adopted [55]. In the EFA, the principal axis factor analysis method was adopted and the Promax method was used for factor rotation [56]. In this phase, items with factor loadings less than 0.40, cross-loadings, and communality less than 0.40 need to be deleted [57].

The study adopted AMOS 20.0 to conduct confirmatory factor analysis (CFA) and cross-group consistency test and SPSS 24.0 to conduct reliability and validity tests on the data from the second phase. In the CFA, χ^2^/df < 3, RMSEA (Root mean square error of approximation) < 0.08, CFI (Comparative fit index), IFI (Incremental fit index), TLI (Tucker-lewis index) > 0.90, PNFI (Parsimonious normed fit index) and PCFI (Parsimonious comparative fit index) > 0.50, were used as the criteria for good model fit [58]. In the reliability test, the Cronbach’s α coefficient and split-half reliability of the scale were calculated. The study takes 0.70 as an indicator of good reliability [59]. In addition, in order to examine whether PS has cross-gender invariance, the study constructs four models, including the configural invariance model (M1), weak invariance model (M2), strong invariance model (M3), and strict invariance model (M4) [60]. The differences of CFI and RMSEA between M2 and M1, M3 and M2, M4 and M3 were compared in turn. If ∆CFI and ∆RMSEA are less than 0.01, then PS is consistent across groups [61].

## Results

### Item analysis

In item analysis(see Table 1), the independent sample t-test was first adopted to calculate the difference between the items in the high-score group (top 27% of the total score) and the low-score group (bottom 27% of the total score). Grouping with the 27% standard can improve the sensitivity and precision of the item-discrimination index, and is the most commonly used method in extreme group division [62]. The results showed that the scores of each item in the high group were significantly higher than those in the low group (*t* = 13.67 to 18.93). Secondly, Pearson correlation analysis was used to calculate the correlation between each item and the total score, so as to investigate the homogeneity of items. The results showed that the item-total correlation coefficient ranged from 0.72 to 0.83. Thirdly, the Cronbach’s α coefficient value of PS was calculated as 0.919. After deleting any item, if the Cronbach’s α coefficient value of the scale increases, it indicates that the homogeneity of the item is poor. However, the results showed that Cronbach’s α coefficient of the scale was lower than 0.919 (0.905 to 0.915) for each item deleted, so all items were preserved in the item analysis.

**Table 1:** Item analysis results of PS

Item	Whole Sample (*N* = 302)		Low Group (*N* = 82)		High Group (*N* = 82)		t value	Correlation Coefficients	α if Deleted
	M	SD	M	SD	M	SD			
1	3.13	1.04	2.16	0.75	3.99	0.91	14.09^***^	0.75^***^	0.913
2	3.26	1.03	2.29	0.82	4.13	0.68	15.60^***^	0.76^***^	0.911
3	3.39	1.01	2.49	0.92	4.22	0.69	13.67^***^	0.74^***^	0.913
4	3.21	1.05	2.04	0.78	4.18	0.69	18.74^***^	0.82^***^	0.906
5	3.22	0.94	2.32	0.80	4.04	0.68	14.88^***^	0.79^***^	0.909
6	3.26	1.03	2.34	0.85	4.16	0.62	15.66^***^	0.72^***^	0.915
7	3.18	1.08	2.12	0.76	4.22	0.69	18.56^***^	0.81^***^	0.908
8	3.08	1.06	1.94	0.73	4.04	0.69	18.93^***^	0.80^***^	0.909
9	3.07	1.03	2.05	0.70	4.09	0.72	18.31^***^	0.83^***^	0.905

^***^p < 0.001; *M* Mean, *SD* Standard Deviation

### Exploratory factor analysis (EFA)

The value of KMO of the scale was 0.937, and Bartlett’s test of sphericity was 1548.95 (df = 36, *p*<0.001), suggesting that the scale is suitable for EFA. In EFA(see Table 2), only one factor was extracted, which could explain 60.91% of the total variance. In addition, all items meet the retention criteria, and the factor loadings ranged from 0.67 to 0.82, with communalities from 0.44 to 0.68. According to EFA results, the study retained all items and was consistent with the original scale in terms of dimension division.

**Table 2:** EFA results of PS (*N* = 302)

Item	Factor Loading	Commonality
Item 1 When I find time for myself, I succeed better at making future plans.	0.70	0.50
Item 2 I like carving out time to enjoy being by myself in a pleasant place/environment.	0.72	0.52
Item 3 I enjoy carving out time for myself to look outside my house or gaze at the scenery.	0.69	0.48
Item 4 When I find time for myself, I feel focused and enable to achieve my best results.	0.80	0.65
Item 5 When I am by myself, I can achieve the high level of focus that I need.	0.77	0.60
Item 6 When I am stressed, having time by myself helps me clear my mind.	0.67	0.44
Item 7 Time for myself gives my life more meaning.	0.78	0.61
Item 8 Time for myself enhances my creativity.	0.77	0.59
Item 9 Finding time for myself contributes to my quality of life.	0.82	0.68

### Confirmatory factor analysis (CFA)

It is demonstrated by the CFA results that the model fits well with each fit indices of χ^2^/df = 2.771, RMSEA = 0.076, CFI = 0.972, IFI = 0.972, TLI = 0.959, PNFI = 0.665, PCFI = 0.675.

### Test of criterion-related validity

Pearson correlation analysis results showed (see Table 3) that PS scores were significantly positively correlated with SWLS, Autonomy, Competence, and Relatedness scores (*r* = 0.45 to 0.44). PS scores were significantly negatively correlated with ULS-6, K10, EDS, and AAQ-II scores (*r*= -0.27 to -0.36).

**Table 3:** Criterion Validity Test of the PS (*N* = 306)

	1	2	3	4	5	6	7	8	9
1.PS	-								
2.ULS-6	-0.30^**^	-							
3.SWLS	0.44^**^	-0.50^**^	-						
4.Autonomy	0.38^**^	-0.45^**^	0.71^**^	-					
5.Competence	0.39^**^	-0.47^**^	0.70^**^	0.76^**^	-				
6.Relatedness	0.35^**^	-0.53^**^	0.67^**^	0.70^**^	0.76^**^	-			
7.K10	-0.28^**^	0.65^**^	-0.44^**^	-0.44^**^	-0.46^**^	-0.47^**^	-		
8.EDS	-0.27^**^	0.59^**^	-0.35^**^	-0.29^**^	-0.28^**^	-0.30^**^	0.73^**^	-	
9.AAQ-II	-0.36^**^	0.59^**^	-0.40^**^	-0.30^**^	-0.27^**^	-0.33^**^	0.58^**^	0.69^**^	-
M	29.05	12.64	23.68	14.37	14.83	15.22	24.70	12.60	24.42
SD	7.08	3.23	5.65	3.48	3.58	3.57	8.06	4.20	8.13

^**^p < 0.01;*PS* Positive Solitude Scale, *ULS-6* Short-Form UCLA Loneliness Scale, *SWLS* Satisfaction With Life Scale, *K10* Kessler Psychological Distress Scale, *EDS* Ego Depletion Scale, *AAQ-II* Acceptance and Action Questionnaire-2nd Edition, *M* Mean, *SD* Standard Deviation

### Reliability test

The reliability test was performed using the data from the second phase. The results showed that the Cronbach’s α coefficient value of PS was 0.917, and the split-half reliability value was 0.928, both of which were greater than 0.70, indicating that PS has a good reliability.

### Cross-gender invariance test

The results indicated (see Table 4) that the four models had good fits, which meets the precondition of the cross-group invariance test. In the comparison between M2 and M1, M3 and M2, and M4 and M3, ∆CFI is 0.002, 0.005, and 0.004 respectively. ∆RMSEA is -0.006, -0.007, and − 0.006, respectively. These results indicated that PS has cross-gender equivalence.

On this basis, the study further examined gender differences in positive solitude. The *t* test results of independent samples showed that there was no significant difference in PS scores between older men (29.34 ± 7.90) and older women (28.84 ± 6.46) (*t* = 0.59, *p* = 0.56).

**Table 4:** Cross-gender invariance test results of PS (*N* = 306)

Model	χ^2^	df	χ^2^/df	CFI	IFI	TLI	RMSEA (90%CI)	∆CFI	∆RMSEA
M1	99.87	50	1.997	0.968	0.968	0.954	0.057 (0.041∼0.074)		
M2	103.94	58	1.792	0.970	0.971	0.963	0.051 (0.035∼0.067)	0.002	-0.006
M3	106.67	67	1.592	0.975	0.975	0.973	0.044 (0.028∼0.059)	0.005	-0.007
M4	109.14	76	1.436	0.979	0.979	0.980	0.038 (0.020∼0.053)	0.004	-0.006

*M1* Configural Invariance model, *M2* Weak Invariance model, *M3* Strong Invariance model, *M4* Strict Invariance model, *RMSEA* Root mean square error of approximation, *CFI* Comparative fit index, *IFI* Incremental fit index, *TLI* Tucker-lewis index

## Discussion

PS was originally developed among the middle-aged and older population in Israel. However, the Chinese version of the Positive Solitude Scale (PS) was adopted for only college students and the middle-aged population [36, 37]. The current study examined the psychometric characteristics of PS in older Chinese people, whose number accounts for 14.9% of the total population in this country [63]. This measurement of positive solitude available for older adults can be propitious to be used in studies of older people’s mental health These studies can be equipped with a valid tool to investigate the effect of positive solitude on reducing negative outcomes (i.e., loneliness, daily stress) and improving positive outcomes (i.e., quality of life) [64]. Therefore, it would be important to investigate whether positive solitude may mediate or moderate adverse implications caused by poor health, loss of a spouse, living alone, reduced social support, and dwindling social interaction for older people [65].

Item analysis, as one of the fundamental evaluations of the quality of the item to judge whether the item can be retained, was not performed in the original scale study. Therefore, the item analysis of the Chinese version of PS was further conducted in this study. It was found that the scores of PS items in the high-group were significantly higher than those in the low-group, indicating that each item can distinguish different levels of positive solitude well. In addition, the item-total correlation coefficient was greater than 0.40, and after deleting any item, the Cronbach’s α coefficient value of the scale decreased to different degrees. The results showed that the Chinese version of PS had a high positive correlation among the items, and measured the same construct. Based on the item analysis, the research performed the factor analysis. One factor was extracted through EFA, which could explain 60.91% of the total variance. Further CFA showed that the fitting index of the single-factor model was good. The results of factor analysis showed that the Chinese version of PS had good construct validity.

The unidimensional structure explored in this study was consistent with the original scale [32], and was also consistent with the research results in the young and middle-aged population in China [36]. This study once again verified the rationality of the single-factor structure scale in the older population in China. However, it is worth noting that it is unclear whether the unidimensional structure adequately reflected the full connotations of the construct of positive solitude which is considered to be a complex concept that is made up of multiple components. In the qualitative study of Mor et al., it was pointed out that positive solitude was composed of 7 content categories and 3 meta-themes [13] like “positive solitude is a choice”; “positive solitude leads to satisfaction and happiness”; and “positive solitude makes sense”. This definition has been adopted in most subsequent quantitative studies on positive solitude, especially in the development of PS. Moreover, it was emphasized on most scales related to solitude, for example, Preference for Solitude Scale (PSS) [23], Solitude Questionnaire (SQ) [25], Motivation for Solitude Scale - Short Form (MSS-SF) [27], Children’s Solitude Scale (CSS) [29], and Child Social Preference Scale (CSPS) [30], that solitude is composed of multiple dimensions. Therefore, whether the unidimensional structure is the optimal structure of PS still needs more research and in-depth discussion.

The results of criterion validity analysis showed that the scores of PS were significantly positively correlated with those of each dimension of the Satisfaction With Life Scale (SWLS) and Need Satisfaction Scale (NSS), and significantly negatively correlated with those of the Short-Form UCLA Loneliness Scale (ULS-6), the Kessler Psychological Distress Scale (K10), the Ego Depletion Scale (EDS), and the Acceptance and Action Questionnaire-2nd Edition (AAQ-II). The results of this study showed that positive solitude is associated with meeting individual psychological needs, such as mental flexibility, reduced loneliness and self-depletion, and better mental health. Previous studies have found that solitude is a complex multi-dimensional structure and believed that self-determined positive solitude can promote individual mental health and self-development, while constrained negative solitude may bring negative impacts [38, 66]. This study also suggests that positive solitude is a meaningful and valuable activity for older adults, associated with positive mental health outcomes.

The study examined the cross-group invariance of PS. The results showed that PS was equivalent in male and female older people, which met the prerequisite for gender comparison. The results of further difference analysis showed that the score of positive solitude of older men was higher than that of older women, but there was no significant difference. In the study of Yu et al., it was found that women’s level of positive solitude was higher than that of men [36]. However, this study showed that there were no significant gender differences in the experience of positive solitude for older Chinese people. The age difference may be one of the underlying reasons. In the study of Yu et al., the main participants were under 30 years old, while the participants of this study were older people over 65 years old. Moreover, Yu et al. surveyed during the recurrent outbreak of COVID-19, which resulted in policies limiting travel and controlling social distancing taken by the Chinese government.

Ost-Mor et al. found no significant correlation between gender and positive solitude in their study among older adults [67]. The current study concluded the consistent result that no significant gender difference in positive solitude. The reason may be that older adults benefit more due to positive solitude and their more positive attitude. Pavlidis et al. highlighted in study that the majority of older adults whether male or female are satisfied with their solitude, which shows no significant gender difference in satisfaction with positive solitude [68]. By comparing with other age groups, older adults experience solitude more positively and can benefit from solitude-seeking [8]. Positive solitude contributes to the improvement of inner quietness and sense of pleasure for older adults, which is conducive to better dealing with loneliness [13, 67]. It is precisely because positive solitude promotes the better adaptation of older people to later life that older adults of different genders show higher levels of positive solitude.

This study has certain theoretical significance as the first to test the reliability and validity of PS in older Chinese population. Cultural factors can affect individuals’ attitudes towards solitude and their behavioral responses when they are alone [34]. The results showed that the Chinese version of PS showed satisfactory psychometric characteristics in the older population, indicating that PS has a certain cross-cultural applicability. The results of this study can provide a tool to support the cross-cultural comparative study of positive solitude. In addition, through the analysis of criterion validity, this study helps to better reveal the positive role of positive solitude in individual development.

This study also encompasses some practical value. Improving older adult’s ability to positive solitude will help them better adapt to life in old age. The Chinese version of PS can provide instrumental support for evaluating the extent of older adults’ positive solitude and identifying the target group. In addition, China’s population aging process is accelerating, and the proportion of older people in the total population is increasing. What’s more, aging is a universal and global phenomenon. How to improve the mental health and well-being of older adults, so as to assure them a better enjoyed old age is one of the responsibilities of governments. The cultivation of the positive solitude ability of older adults is conducive to the realization of the above goals.

### Limitations and future research

There are some limitations in this study that deserve concern in future studies. First, the study adopted the method of convenient sampling, which may cause the problem of under-representation of samples. Meanwhile, the study did not examine the test-retest reliability of PS, and it is not yet possible to know whether the scale has cross-time stability in older population in China. Second, the study did not examine the validity of PS in populations with different demographic characteristics. Age, health, living environment, culture, and marital status are all potential influencing factors for the positive solitude of older adults [10]. Therefore, in future studies, it is necessary to further analyze the reliability and validity of the scale in different older groups with various demographic characteristics. For instance, examining the validity of PS in older adults who are widowed, disabled, living alone, suffering from serious diseases, or very old. Third, positive solitude generally exists in people of different ages, and this study did not test the validity of PS in middle-aged people, adolescents, or children.

## Conclusion

Solitude is an important part of older people’s daily lives and a key factor in health and well-being. Individuals voluntarily or actively choosing to be alone and enjoying being alone can have a positive impact on their quality of life. To this end, the current study examined the validity of PS in older populations to provide instrumental support. It was found that the Chinese version of PS consists of 9 items, which is a unidimensional structure, consistent with the original scale. In addition, PS has good reliability and validity in the older Chinese population and can be used as a reliable and concise tool to evaluate the positive solitude of older Chinese adults.

## Data Availability

No datasets were generated or analysed during the current study.

## Abbreviations

PS: Positive solitude scale
PSS: Preference for solitude scale
SWLS: Satisfaction with life scale
ULS-6: Short-form UCLA loneliness scale
K10: Kessler psychological distress scale
EDS: Ego depletion scale
AAQ-II: Acceptance and action questionnaire-2nd Edition
SQ: Solitude questionnaire
SolAS: Aloneliness scale
MSS-SF: Motivation for solitude scale - short form
SDS: Self-determined solitude
NSDS: Not self-determined solitude
CSS: Children’s solitude scale
CSPS: Child social preference scale
NSS: Needs satisfaction scale
EFA: Exploratory factor analysis
CFA: Confirmatory factor analysis
M1: Configural invariance model
M2: Weak invariance model
M3: Strong invariance model
M4: Strict invariance model
M: Mean
SD: Standard deviation
